# Distinct Tumor Microenvironment at Tumor Edge as a Result of Astrocyte Activation Is Associated With Therapeutic Resistance for Brain Tumor

**DOI:** 10.3389/fonc.2019.00307

**Published:** 2019-04-26

**Authors:** Chiu-Min Lin, Ching-Fang Yu, Hsueh-Ya Huang, Fang-Hsin Chen, Ji-Hong Hong, Chi-Shiun Chiang

**Affiliations:** ^1^Department of Biomedical Engineering and Environmental Sciences, National Tsing Hua University, Hsinchu, Taiwan; ^2^Department of Radiation Oncology, Chang Gung Memorial Hospital Linkou Branch, Taoyuan, Taiwan; ^3^Radiation Biology Research Center, Institute for Radiological Research, Chang Gung Memorial Hospital, Chang Gung University, Taoyuan, Taiwan; ^4^Education & Medical Research National Taiwan University Hospital Hsin-Chu Branch, Hsinchu, Taiwan; ^5^Department of Medical Imaging and Radiological Sciences, Chang Gung University, Taoyuan, Taiwan; ^6^Institute of Nuclear Engineering and Science, National Tsing Hua University, Hsinchu, Taiwan; ^7^Frontier Research Center on Fundamental and Applied Sciences of Matters, National Tsing Hua University, Hsinchu, Taiwan

**Keywords:** brain tumor, tumor hypoxia, peripheral hypoxia, therapeutic resistance, astrocyte, tumor recurrence

## Abstract

Tumor vasculatures and hypoxia are critical tumor micro-environmental factors associated with tumor response to the therapy and heterogeneous in both time- and location-dependent manner. Using a murine orthotopic anaplastic astrocytoma model, ALTS1C1, this study showed that brain tumor edge had a very unique microenvironment, having higher microvascular density (MVD) and better vessel function than the tumor core, but on the other hand was also positive for hypoxia markers, such as pimonidazole (PIMO), hypoxia inducible factor-1α (HIF-1α), and carbonic anhydrase IV (CAIX). The hypoxia at tumor edge was transient, named as peripheral hypoxia, and caused by different mechanisms from the chronic hypoxia in tumor core. The correlation of CAIX staining with astrocyte activation marker, glial fibrillary acid protein (GFAP), at the tumor edge indicated the involvement of astrocyte activation on the development of peripheral hypoxia. Peripheral hypoxia was a specific trait of orthotopic brain tumors at tumor edge, regardless of tumor origin. The hypoxic cells were resistant to the therapy, regardless of their location. Surviving cells, particularly those at the hypoxic region of tumor edge, are likely the cause of tumor recurrence after the therapy. New therapeutic platform that targets cells in tumor edge is likely to achieve better treatment outcomes.

## Introduction

High-grade gliomas such as anaplastic astrocytoma and grade IV glioblastomas remain as lethal cancers even after aggressive treatment. The median survival rate for gliomas is <15 months and 5-year survival rate is only around 5% (1). Complete tumor resection is very difficult to achieve because wide resection margin is usually not allowed in the brain and tumor is very infiltrative into normal brain tissue. Post-operative radiotherapy is essential and combination with Temozolomide (TMZ) was shown to prolong survival time (2). Although combination of chemotherapy and radiotherapy becomes the current standard of care, this is not able to achieve substantial cure in the long run. Many therapeutic trials had been tested in the clinics, but very few were shown to significantly prolong patients' survival.

Local recurrence is the main site of treatment failure and usually occurs within less than a year after treatment (3), suggesting high-grade glioma is an aggressive tumor and resistant to radiotherapy and chemotherapy. The molecular mechanisms for their behaviors and responses to treatment are not fully elucidated. Molecular markers such as epidermal growth factor receptor (EGFR) amplification (4), isocitrate dehydrogenase (IDH) 1/2 mutation (5), and O6-methylguanine-DNA-methyltransferase (MGMT) promoter methylation (6) were shown to related to clinical behaviors and responses to anti-angiogenesis and chemotherapy. However, markers predicting tumor response to radiotherapy are lacking; this is because the mechanisms for radioresistance of high-grade glioma are complex and multifactorial, and there is spatial and temporal limitation of getting human brain tumor tissue during and after the treatment.

Based on the *in vitro* assay of survival fraction at 2 Gy (7) and *in vivo* assay in laboratory animals (8), the intrinsic radiosensitivity of malignant gliomas is in a wide range and not correlated with clinical outcome. This suggests other factors are associated with radioresistance in malignant gliomas. Many studies have shown that a small portion of tumor cells, which means cancer stem cell in glioblastoma contribute the radioresistance (9, 10). Furthermore, the radioresistance is also through the interaction between cancer stem cell and tumor microenvironment (11).

It is well-known that tumor microenvironment plays an important role in tumor behaviors and responses to treatment, but the tumor microenvironment in high-grade gliomas such as GBM is very heterogeneous. One of the biological characteristics in high-grade glioma is highly invasion into surrounding normal brain tissue, but migration and proliferation of glioma cells are mutually exclusive (so call “go or grow”) (12) and therefore the tumor microenvironment in the central and edge (invasion part) should be different. We have set up a murine orthotopic astrocytoma model, ALTS1C1, and found it is very similar to human high-grade glioma with infiltration to adjacent normal brain tissue (13). By using this tumor model, we found that tumor-secreted stromal cell-derived factor-1 (SDF-1) is associated with invasiveness of glioma cells (13) and tumor invasion after radiotherapy (14). These findings were subsequently confirmed in human glioma cells (15) and in human glioma tumor tissues taken before and after radiotherapy (16), suggesting the behaviors in our animal model are reproducible in human. We also found the tumor microenvironment, in terms of the number of tumor-associated macrophages, microvascular density, and expression of matrix metalloproteinase-2 (MMP-2), in the recurrent tumor after radiotherapy is different between primary tumor core and tumor edge (14, 17).

In the present study, we used same murine orthotopic astrocytoma model to further explore the differences of tumor microenvironment in the primary tumor core and edge (invasion part). We focus on the vascular density and structure, hypoxia status, and responses to cytotoxic treatment. Since hypoxia and vascular damage play important roles in the responses of radiotherapy and chemotherapy, this study provides important information in the distinct tumor microenvironment in the tumor edge and clues for designing better treatment strategy in high-grade astrocytoma.

## Materials and Methods

### Mice

Male C57BL/6J mice on age of 8 weeks were purchased from the National Laboratory Animal Center (NLAC), Taipei, Taiwan. All animal experimental procedures were complied with the guideline approved by the Institutional Animal Care and Use Committee (IACUC) of National Tsing Hua University, Taiwan (IACUC: 10145).

### Cell Line Culture

A murine astrocytoma cell line, ALTS1C1 (BCRC60582, Hsin-Chu, Taiwan), was originally established in our lab (13). Murine glioma cell GL261 was obtained from Dr. Newcomb's Lab (18). Murine melanoma cell line B16-F0, was purchased from ATCC (CRL-6322, Manassas, VA, USA). Cells were maintained in Dulbecco's modified Eagle's medium (GIBCO, Thermo Fisher Scientific, Inc., Waltham, MA, USA) with 10% fetal bovine serum (GIBCO) and 1% penicillin-streptomycin (GIBCO), and incubated with a humidified 5% CO_2_/95% air atmosphere at 37°C.

### Tumor Implantation and Treatments

For intracranial (I.C.) implantation, 1 × 10^5^ tumor cells were intracranially inoculated into mice. The protocol of I.C. tumor inoculation was described in detail in previous publication (13). Tumor-bearing mice were sacrificed at designed day. For intramuscular (I.M.) tumors and subcutaneous (S.C.) tumors, 3 × 10^6^ tumor cells were intramuscularly injected into the right thigh or subcutaneously into the right flank, respectively, of C57BL/6J mice. Tumor sizes were measured daily with a caliper until the experiment was completed.

After 14 days with tumor inoculation, brain tumor-bearing mice were given the whole brain irradiation according to the protocol developed in previous studies (14, 19). Briefly, mice were irradiated under the anesthetization by 6-MV X-ray from a linear accelerator, and the dose rate for irradiation was around 6 Gy/min. The field size of irradiation was 1 cm (the region behind the eye and ahead of the ears) and the mice were covered with 1 cm bolus during the irradiation. For chemotherapy, mice were injected intraperitoneally (i.p.) with PBS or TMZ (50 mg/kg, Sigma, St. Louis, MO, USA) at day 14 of tumor inoculation, and sacrificed 24 h after TMZ treatment.

### Assessment of Hypoxic Region

Tumor hypoxia was examined by i.p. administrating pimonidazole (PIMO, 160 mg/kg, HP1-100kit, HPI, Burlington, MA, USA) into mice 1 h before mice sacrificed. Tissues embedded in OCT compound (Sakura Finetek, Torrance, CA, USA) were kept in −80°C. To explore the dynamic change of hypoxia in tumors, two hypoxia markers, PIMO and CCI-103F (160 mg/kg, HP4-100kit, HPI), were given at different time points to define hypoxic regions. Mice were i.p. injected PIMO and CCI-103F 5 and 1 h, respectively, before sacrifice.

### Vascular Function Analysis

Tumor-bearing mice were intraveneously (i.v) injected tetramethylrhodamine 70 kDa dextran (80 mg/kg, Molecular Probes, Waltham, MA, USA) or DyLight 594 labeled lycopersicon esculentum (Tomato) lectin (4 mg/kg, Vector Laboratories, Burlingame, CA, USA), and sacrificed after 40 and 2 min, respectively (20). Leakage index was defined as the ratio of the signals (pixels) of 70 kDa-dextran to the number of CD31+ vessels.

### Immunohistochemical (IHC) Analysis

Frozen sections were fixed in cold 100% methanol for 5 min. For immunoperoxidase staining, sections were incubated with 0.1–1% hydrogen peroxide/PBS for 5–10 min at room temperature (RT) to inhibit endogenous peroxidase activity. After wash, sections were mounted with PBS containing 4% FBS, 1% normal goat serum (GIBCO), 0.01% Tween-20 (Sigma) and 0.1%Triton (Sigma) for 1 h at RT to avoid non-specific binding. Frozen sections were subsequently stained with specific primary antibody against rat anti-mouse CD31 (1:200), rabbit anti-mouse caspase-3 (1:500) (both from BD Phamingen, San Jose, CA, USA), mouse anti-mouse GFAP (1:1000, Sigma), rabbit anti-mouse NG2 (1:200, Millipore, Burlington, MA,USA), mouse anti-mouse HIF-1α (1:100, Novus, Littleton, CO, USA), goat anti-mouse CAIX (1:100, R&D system, Minneapolis, MN, USA), mouse anti-PIMO (1:100), and rabbit anti-CCI-103F (1:100) (both from HPI) and incubated at 4°C overnight. Primary antibodies were detected by secondary antibody conjugated with fluorescent dyes or streptavidin peroxidase (Invitrogen, Waltham, MA, USA) for 30 min at RT. To quantify the staining results and avoid subjective bias, each represented data was scored from the average at least three tumor tissues, for each tissue, at least 3 sections were counted and each section contained at least 10 random fields. Images were processed by Image-Pro Plus 6.0 (Media Cybernetics, Inc., Rockville, MD, USA) and positive cells were counted by ImageJ 1.48v software (NIH, Bethesda, MD, USA).

### Statistics

The statistical analysis was done by GraphPad Prism software version 7 package (GraphPad Software, Inc., San Diego, CA, USA). Difference between treatments group was derived from two-tailed Student's *t*-test or one-way ANOVA and was determined to be statically significant when *P* ≤ 0.05.

## Results

### Differences in Vascular Density and Function Between Tumor Core and Edge

**Figure 1:**
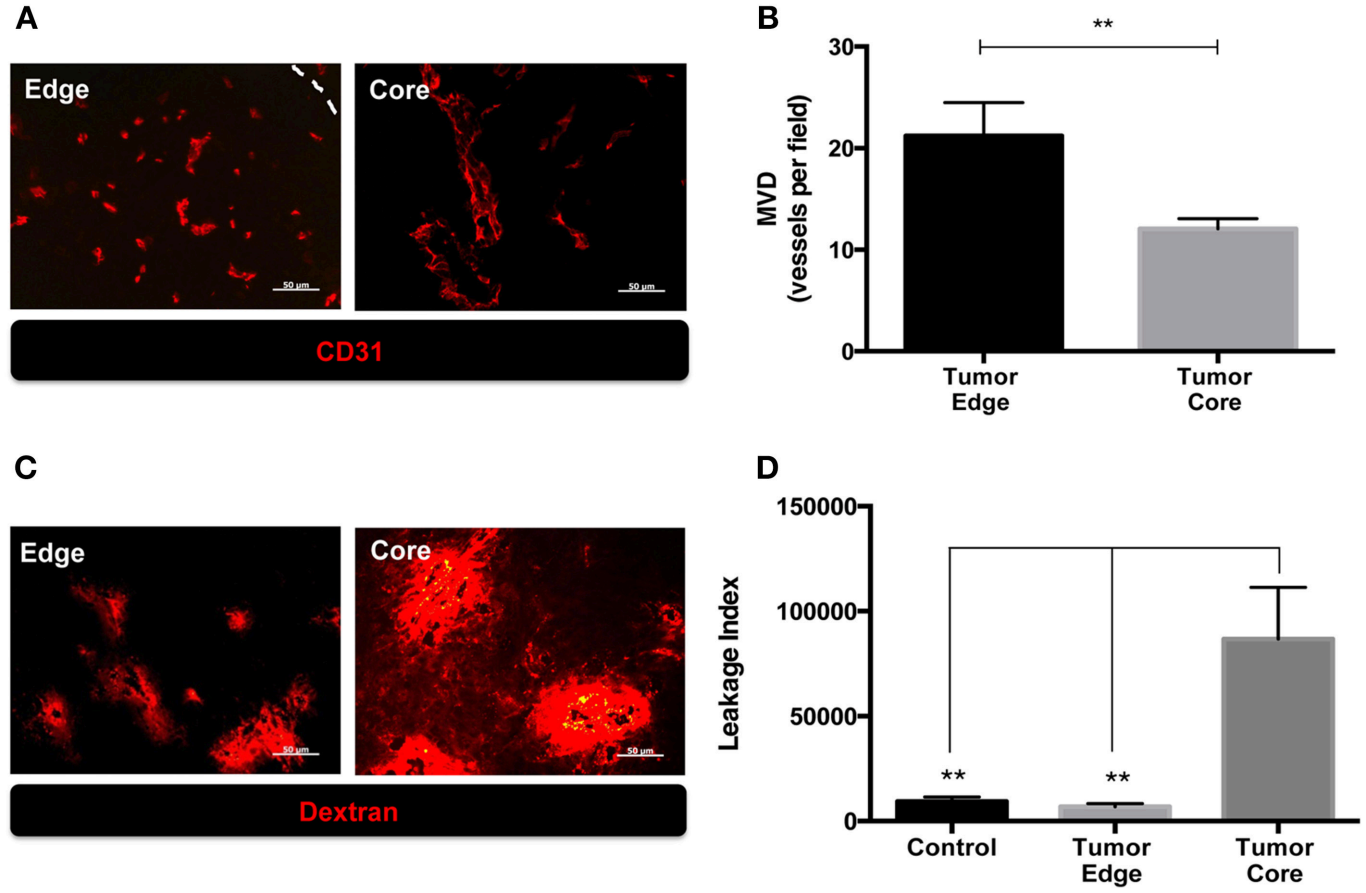
The microenvironment of tumor edge had higher microvascular density as well as better vascular function than those of tumor core in orthotopic ALTS1C1 tumors. Tumor sections at day 21 were stained with endothelial cell marker, CD31, and quantified. **(A)** Representative figures of CD31+ vessels in tumor core and edge. Tumor edge was defined as the distance of 200 μm inward from the tumor's boundary marked by white dotted line. **(B)** MVD was defined as the number of CD31+ vessels per field under 40X magnification. At least 10 fields per section were analyzed. Three sections of each tissue was calculated. Each group had at least three mice for data analysis. **(C)** The integrity of vasculatures in brain tissues injected with PBS (control) and ALTS1C1 tumor cells was examined by 70 kDa dextran distribution. **(D)** Leakage index was defined as the ratio of the signals (pixels) of dextran to the number of CD31+ vessels. All data points were analyzed in triplicate and values displayed are means ± SDs. The significant difference of treatment groups were compared with control group and analyzed by ANOVA. **P < 0.01. Scale bar = 50 μm.

We previously observed the increased number of MMP-2 coupled CD31^+^ cells and CD68+ tumor associated macrophages (TAMs) at the invading front of ALTS1C1 tumors (13, 17). Since these factors affect tumor angiogenesis, in this study we further compared the vascular density, structure and function between tumor core and edge. Mice bearing ALTS1C1 brain tumor began to die in day 21 and median survival time is 24 days (13). As shown in Figures 1A,B, tumor samples were taken from 21 day ALTS1C1 brain tumor and the microvascular density (MVD) in the tumor edge, defined as 200 μm inward from the tumor boundary, was significantly higher than that in tumor core, defined as the tumor region excluded from the edge (*P* = 0.0099). To further explore tumor vessel integrity, fluorescent-labeled 70 kDa dextran was used to measure the leakage index, which was defined as the ratio of the signals of dextran to the number of CD31+ vessels. As shown in Figures 1C,D, the leakage index was significantly higher at the tumor core than the tumor edge (*P* = 0.0012). This indicates that the vessels at the tumor edge have higher density and better function than the vessels in the tumor core. The higher vascular density at tumor edge of this tumor is similar to our previous report (13) showing higher vascular density at invading islands than at tumor core.

### Temporal and Spatial Changes of Hypoxia During Brain Tumor Progression

**Figure 2:**
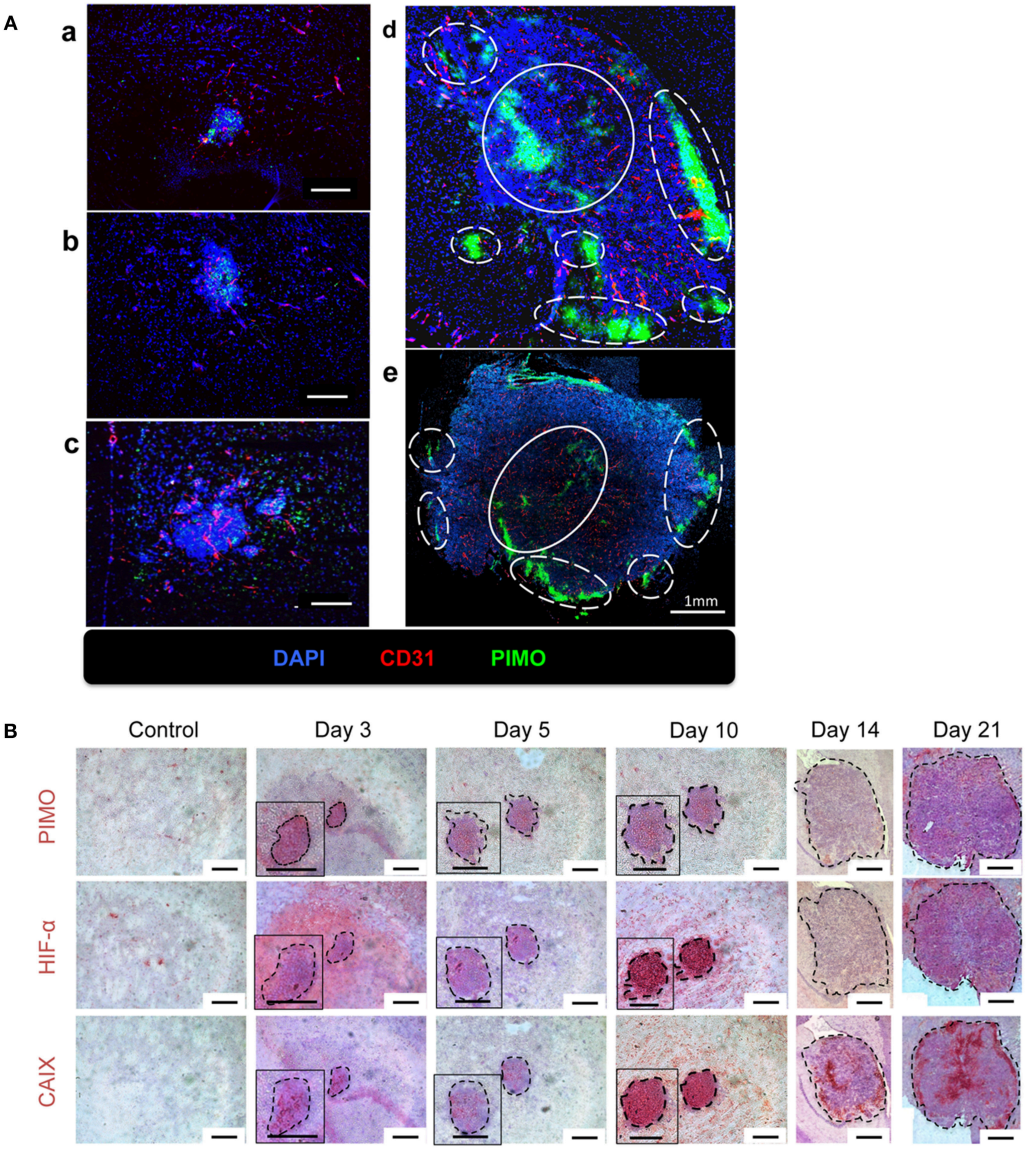
The temporal and spatial pattern of tumor hypoxia-associated markers during tumor progression. **(A)** Mice were sacrificed on day 3 (a), day 5 (b), day 10 (c), day 14 (d), and day 21 (e) after I.C. inoculation of ALTS1C1 tumors. Tumors were double staining for CD31 (red) and hypoxia marker PIMO (green). Nucleus was stained by DAPI (blue). The peripheral hypoxia was marked with white dotted line and the central hypoxia was marked by white solid line. **(B)** Representative figures for hypoxia-associated markers, PIMO (brown), HIF1-α (brown), and CAIX (brown) on normal brain tissue (control) and ALTS1C1 tumor tissue. Nucleus was visualized by staining with hematoxylin (blue) and tumor was outlined by black dotted line. Scale bar = 200 μm.

The above findings suggested that the quantity and quality of tumor vessels are different between tumor core and tumor edge. The hypoxia tracer pimonidazole (PIMO) was then used to examine if these will affect the development of hypoxia during tumor progressions. PIMO-positive hypoxia could be detected as early as 3 days (Figure 2Aa) after intracranial inoculation and was mainly localized in the tumor core at days 5 and 10 (Figures 2Ab,c). As the tumors developed, a second hypoxic region at the tumor edge, termed peripheral hypoxia, became evident at day 14 up to day 21 after tumor inoculation (Figures 2Ad,e). To further characterize and confirm the development of hypoxia, three different hypoxia markers, PIMO, hypoxia-inducible factor-1α (HIF-1α), and carbonic anhydrase IX (CAIX), were utilized (Figure 2B). These markers indicate hypoxia via different mechanisms: PIMO indirectly measures the oxygen concentration within cells (21–23); HIF-1α is a hypoxia-inducible protein and is stabilized under hypoxia; and CAIX is highly expressed by cells under anaerobic metabolism (24). The distribution of these markers during tumor progression (Figure 2B) was as follows: PIMO was the main marker during the early stage (day 3), while HIF-1α and CAIX expression became evident as tumor growth progressed (days 5 to 10). All hypoxic markers were positive on day 10. At later stages (day 14), the hypoxic region could be divided into two major areas, termed peripheral and central hypoxia. The staining pattern for PIMO and HIF-1α were similar, implicating a discontinuous hypoxic rim at the tumor periphery. On the other hand, a more continuous staining pattern for CAIX was observed around the tumor edge (days 14 and 21), which indicated that cells at peripheral regions were actively using anaerobic metabolic pathways.

### Different Mechanisms for Hypoxia at the Tumor Core and Tumor Edge

**Figure 3:**
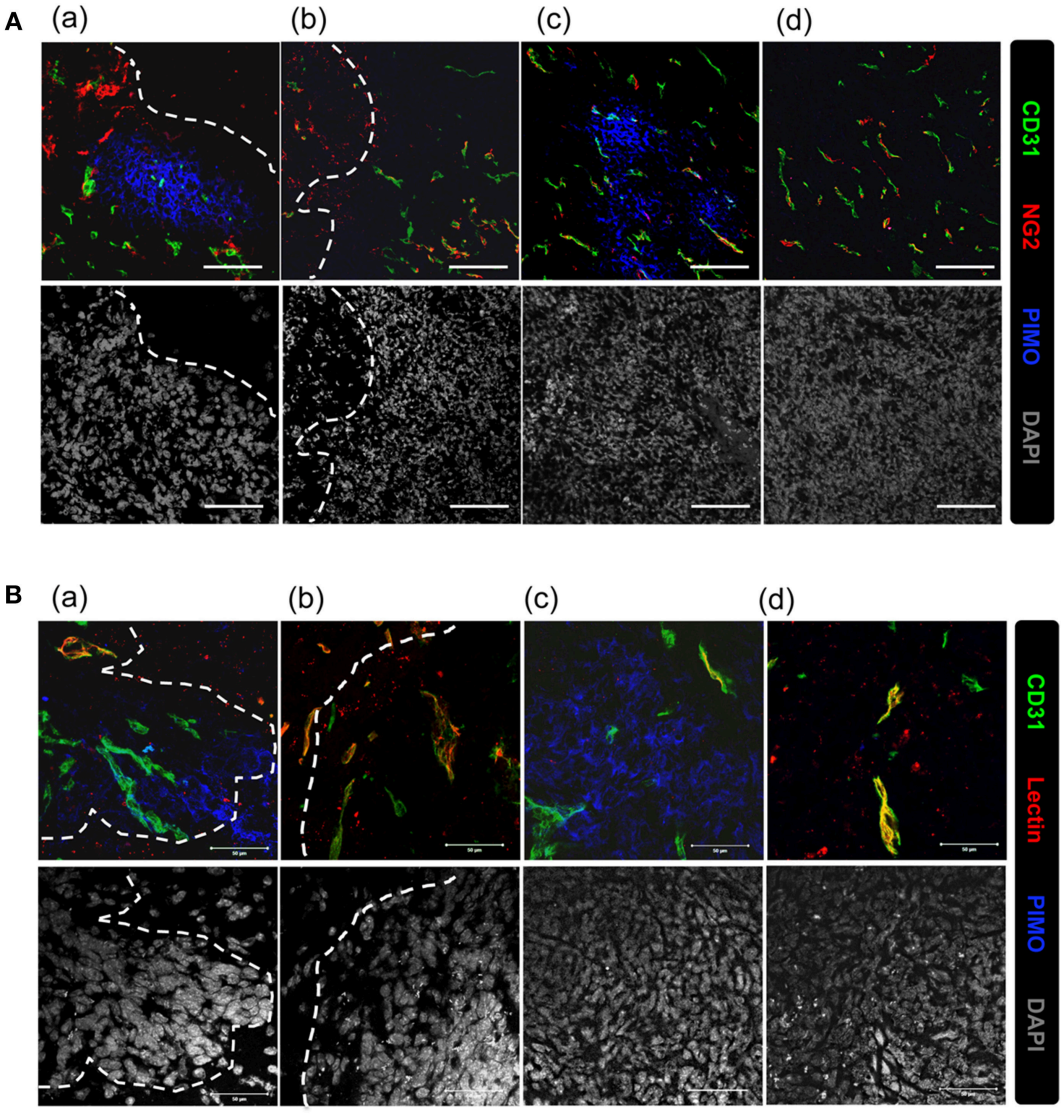
Vascular malfunction, not vascular deficiency, caused the development of peripheral hypoxia. **(A)** Tumor sections were stained for PIMO (blue), CD31 (green), and pericyte marker, NG2 (red), and captured by confocal microscope. Nucleus was stained by DAPI (gray). Scale bar = 100 μm. **(B)** Mice were i.v., injected with lectin (red) before sacrifice, and tissues were co-stained with PIMO (blue) and CD31 (green) for examining blood perfusion at tumor core and edge. Nucleus was stained by DAPI (gray). Tumor boundary was outlined by white dotted line. Scale bar = 50 μm.

Vascular structure in the tumor core and edge was further investigated to explore the reason for inconsistence between the development of hypoxia and higher MVD in tumor edge. Vascular structures were examined for neuron/glial antigen 2 (NG2) and cluster of differentiation 31 (CD31) expression. These two markers were used to identify pericytes and endothelial cells (25, 26), respectively, and co-expression of NG2 with CD31 indicates mature vessels with better perfusion. Additionally, hypoxic regions are indicated by PIMO staining. As shown in Figure 3A, NG2+CD31+ vessels were detected at PIMO-negative regions (Figures 3Ab,d), but not in PIMO-positive regions (Figures 3Aa,c), regardless of location at the tumor edge or at the tumor core. Conversely, more CD31+NG2– vessels were detected in PIMO-positive hypoxic regions at the tumor edge than that of tumor core (Figure 3Aa). Since CD31+NG2– vessels were more prevalent in hypoxic regions at the tumor edge than the tumor core and the lower MVD were measured at tumor core (Figure 1B), a possibility was raised that hypoxia at tumor core and edge is caused by different mechanism; the former is caused by vessel insufficiency (low MVD) while the latter is by vessel malfunction (CD31+NG2– vessels). This hypothesis was explored by retro orbital perfusion with fluorescent-conjugated lectin, which conjugates with endothelial cells and is widely used to study vascular obstruction (20). As shown in Figure 3Ba, most vessels at PIMO-positive hypoxic regions at the tumor edge did not retain the fluorescent lectin tracer (CD31+lectin–), indicating that peripheral hypoxia in tumor edge resulted from vessel malfunction or limited perfusion.

**Figure 4:**
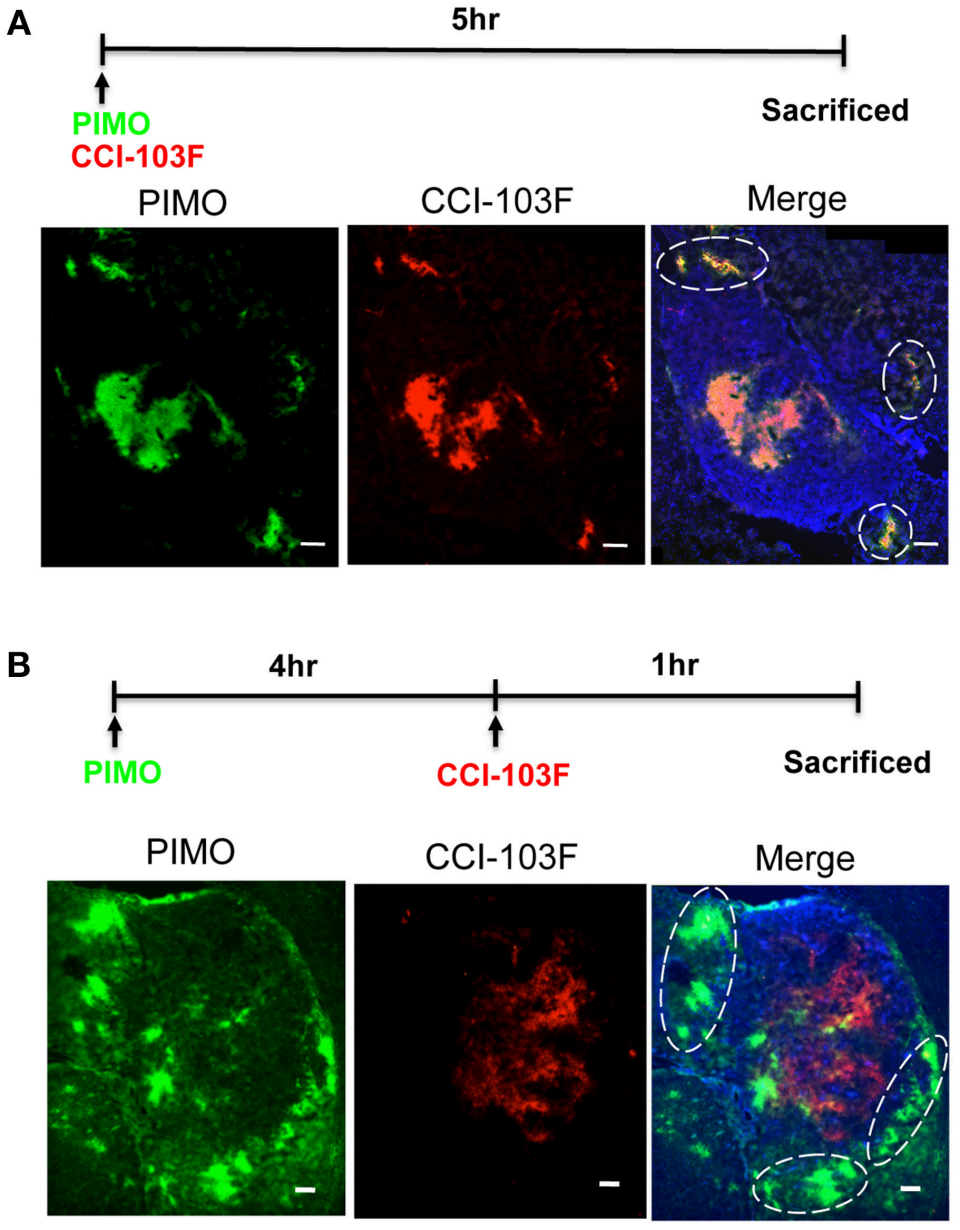
Dynamic shift of hypoxia pattern in ALTS1C1 tumors. The procedure of dual hypoxia tracer was described clearly in the materials and methods section. Dual hypoxia markers, PIMO (green) and CCI-103F (red), were injected simultaneously **(A)** or 5 and 1 h, respectively, before sacrifice **(B)** into mice. The peripheral hypoxia was circled with dotted line. Nucleus was stained by DAPI (blue). Scale bar = 200 μm.

Dual hypoxia tracers, PIMO, and CCI-103F were used to further characterize the temporal nature of hypoxia at tumor edge (27). One group of mice were injected with PIMO and CCI-103F reagents simultaneously, and sacrificed 5 h post-injection. A second group of mice were injected with PIMO, then injected with CCI-103F 4 h later, and then sacrificed 1 h after the last injection. Results show that the patterns of CCI-103F and PIMO were congruent as two reagents were injected simultaneously (Figure 4A), indicating these two agents could stay for at least 5 h. When these two reagents were injected at different time points (Figure 4B), they are not so co-localized as in Figure 4A. The co-localization of both markers in Figure 4B indicates area of chronic hypoxia, which are mainly found in tumor more. On the other hand, the co-localization was not so well at tumor edge, indicating the transient hypoxia at tumor peripheral area.

### A Unique Feature of Hypoxia at Tumor Edge in Tumors Growing in Brain

**Figure 5:**
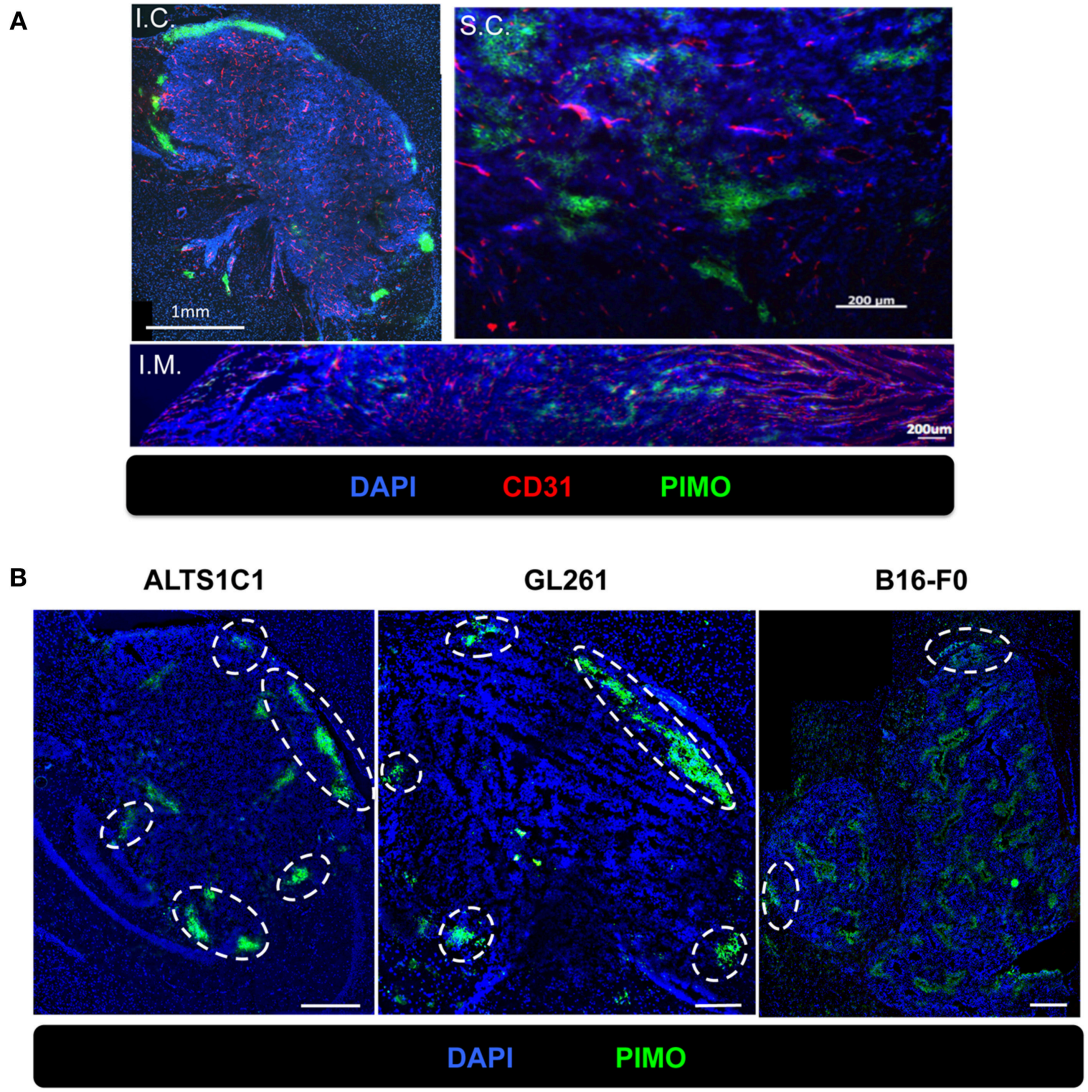
The peripheral hypoxia was a unique phenomenon in orthotopic tumors and independent of tumor types. **(A)** The distribution of hypoxia (green) and vessels (red) was assessed on the ALTS1C1 tumors grown intracranially (I.C.), intramuscularly (I.M.) and subcutaneously (S.C.). Nucleus staining with DAPI was shown in blue. **(B)** Three types of tumor cells, ALTS1C1 (astrocytoma), GL261 (glioma), and B16-F0 (melanoma), were inoculated orthotopically and tumor hypoxia was assessed by PIMO staining. Tumor peripheral hypoxia was circled by white dot line. Scale bar = 400 μm.

To examine if hypoxia at tumor edge is a phenomenon unique to the brain microenvironment, three common tumor inoculation systems including the intracranial (I.C.), intramuscular (I.M.), and subcutaneous (S.C.) models were tested and tissues were examined by IHC staining for PIMO and CD31. The results revealed that tumor hypoxia developed in ALTS1C1 tumors derived from these three models, but peripheral hypoxia was only observed in tumors from I.C. model (Figure 5A), suggesting that peripheral tumor hypoxia is a unique feature of high-grade glioma in brain. To further ensure that the phenomena of peripheral hypoxia found in ALTS1C1 tumors is independent of the tumor origin, three tumor cell lines, astrocytoma (ALTS1C1), glioma (GL261), and melanoma (B16-F0), were inoculated intracranially. Results show that peripheral hypoxia in tumors is a common feature of intracranial tumors and is independent of tumor origin; however, this phenomenon is more apparent in brain-derived cell lines (Figure 5B).

### Activated Astrocytes Were Associated With the Pattern of Peripheral Hypoxia

**Figure 6:**
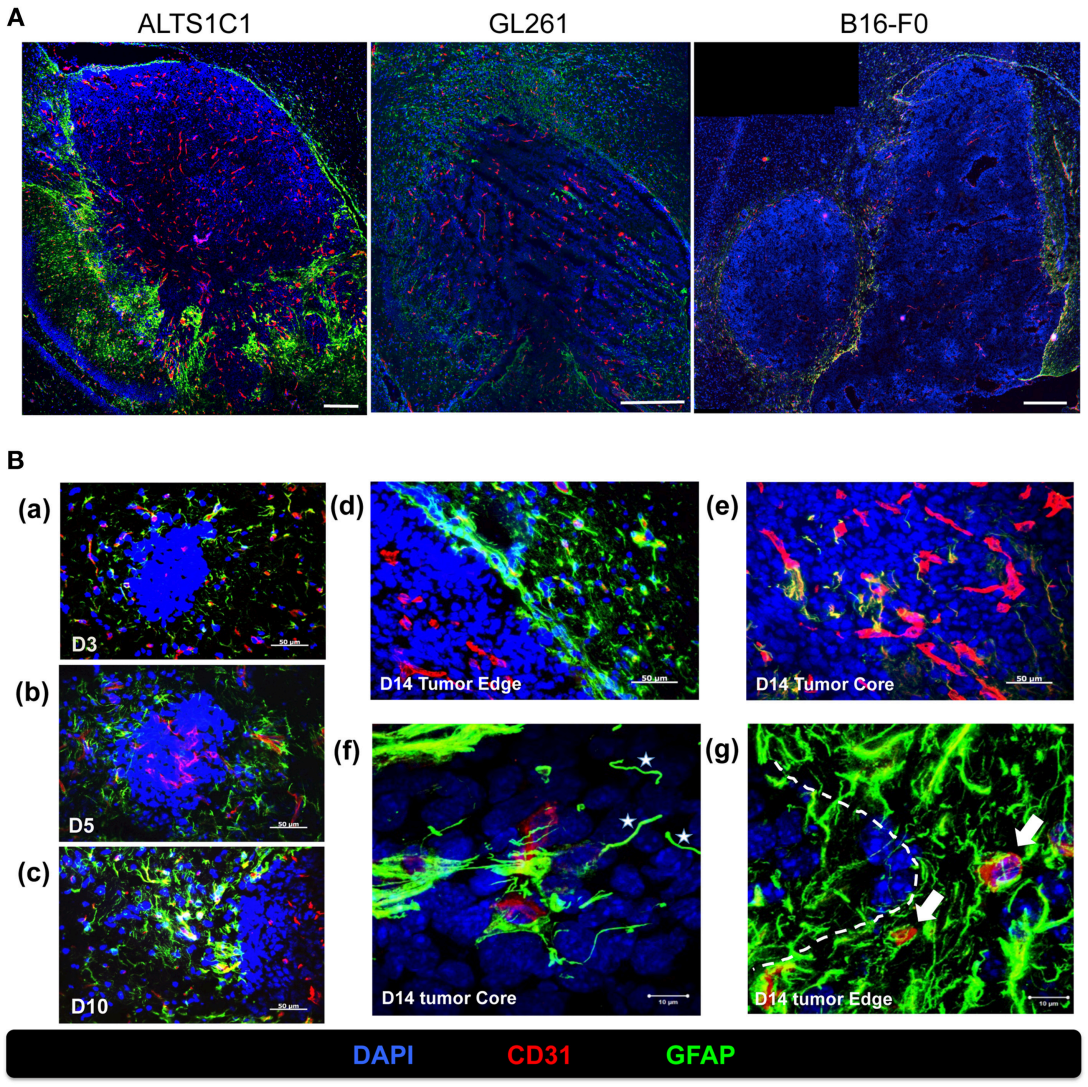
Activated astrocytes surrounded the tumor border during tumor progression. **(A)** The correlation of vasculatures and astrocytes pattern was examined in orthotopic tumors, including astrocytoma ALTS1C1, glioma GL261, and melanoma B16-F0 tumors. Brain tumor tissues were double stained with GFAP (green) and CD31 (red). Scale bar = 400 μm. **(B)** Mice were sacrificed on day 3 (a), day 5 (b), day 10(c), and day 14 (d,e) after I.C. inoculation of ALTS1C1 brain tumor. Tumor sections were double staining for GFAP (green) and CD31 (red). Nucleus was stained with DAPI (blue). Scale bar = 50 μm. (f,g) Representative images at day 14 captured by confocal microscope. White stars marked vessel-like astrocytes (f) and white arrows indicated activated astrocyte-attached vessels (g). Tumor boundary was outlined by white dotted line. Scale bar = 10 μm.

We had demonstrated that peripheral hypoxia in intracranial tumors was brain parenchyma specific and independent of tumor type (Figure 5). It implied that the stromal cells, such as astrocytes and microglia, in brain tissues may be the key role for development of peripheral hypoxia. Molecular marker for astrocytes were assessed by glial fibrillary acidic protein (GFAP) staining and increased expression of GFAP is a frequently used feature of activated astrocytes (28, 29). The results from day 14 after tumor implantation showed that many GFAP^+^ astrocytes activated in the nearby parenchyma of tumor border, and were independent of tumor types (Figure 6A). The intensity of GFAP positive signal was stronger in ALTS1C1 astrocytoma tumors than those in GL261 glioma and B16-F0 melanoma tumors.

To further explore the pattern of activated astrocytes during tumor progression, brain tissues at early stage (days 3, 5, 10) were examined by GFAP staining. Few astrocytes were activated in the parenchyma close to tumors at day 3 after tumor implantation (Figure 6Ba), and astrocytes aggregated gradually when the tumors grew throughout the examination period (Figures 6Bb,c) and formed a sheet-like structure at the tumor edge (Figure 6Bd). The activated astrocytes further invaded the tumor core via vessels (Figure 6Be) and formed a vessel-like scaffold for the adhesion of endothelial cells at day 14 (Figure 6Bf). In addition, activated astrocytes intensively adhered to the vessels in normal tissues closed to the tumors (Figure 6Bg).

**Figure 7:**
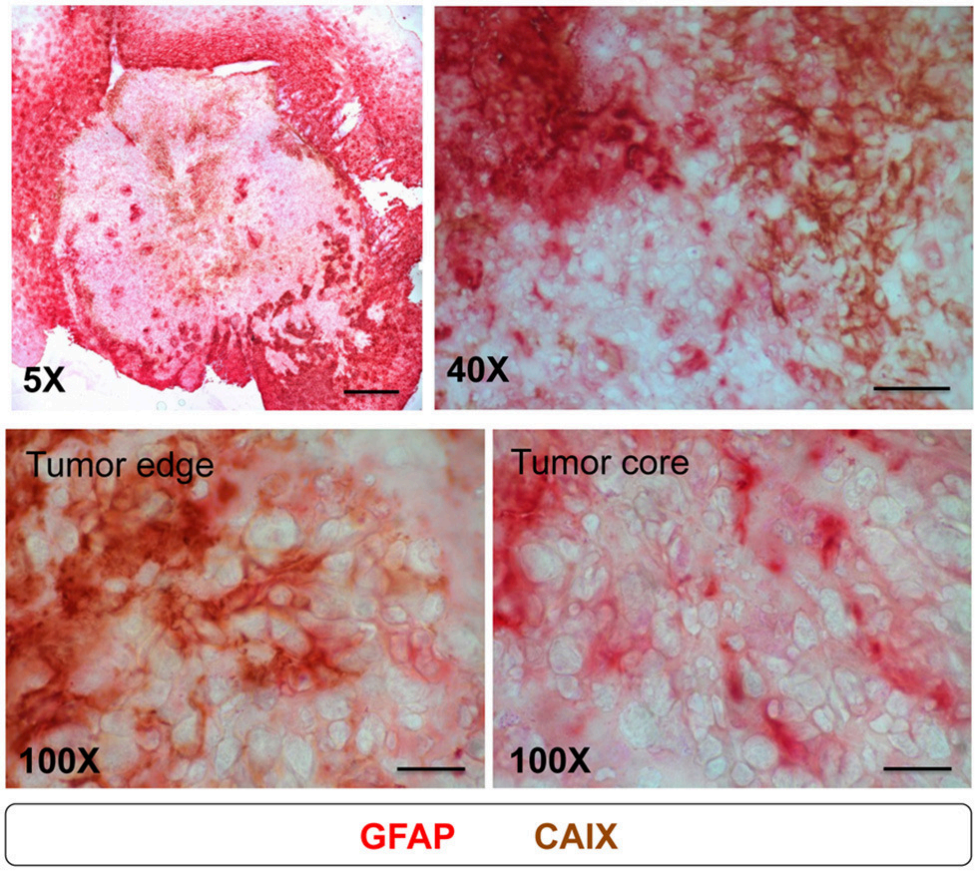
Astrocytes expressed CAIX at tumor edge, but not at tumor core. Mice were sacrificed on day 21 after I.C. inoculation of ALTS1C1 tumor. For examining the correlation of astrocyte and CAIX expression in tumors, sections were double staining by astrocyte marker GFAP (red) and hypoxia-associated marker CAIX (brown). Scale bar = 500 μm for 5X magnification, 50 μm for 40X magnification, and 20 μm for 100X magnification.

Above results (Figure 6) showed that the patterns of activated astrocytes did not correlate with hypoxia at the early stages (days 3 to 10), but had good spatial correlation with peripheral hypoxia at days 14 and 21, particularly with the CAIX staining pattern. To further assess the association of astrocytes and hypoxia, brain tissue at day 21 were co-stained with GFAP and CAIX. The results showed that the signals of GFAP and CAIX were co-localized at tumor edge, but not at tumor core (Figure 7). This suggested that activation of astrocytes might involve in the development of peripheral hypoxia.

### Hypoxia as a Major Reason for Therapeutic Resistance in Tumor Core as Well as Tumor Edge

**Figure 8:**
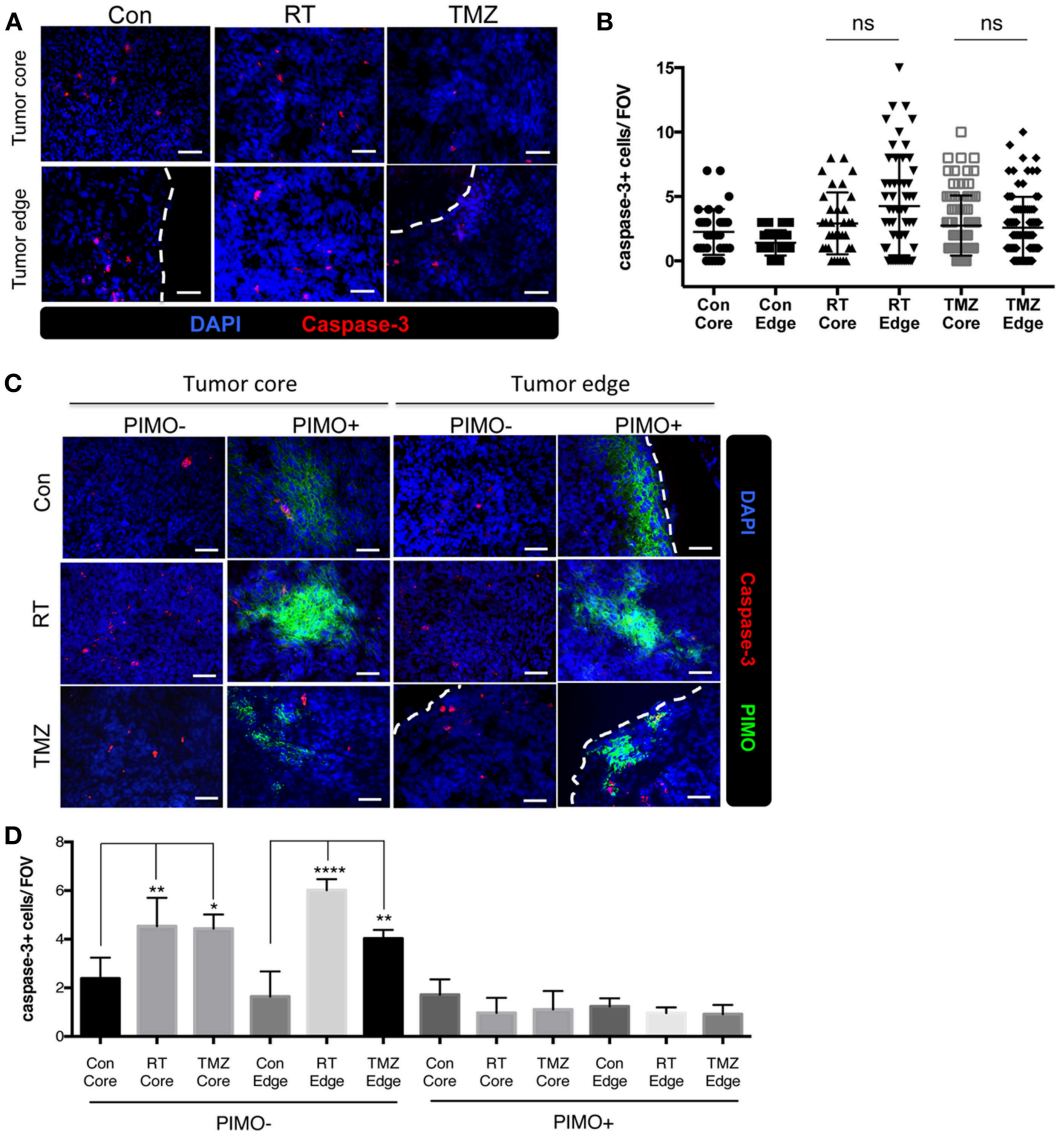
Transient peripheral hypoxia caused brain tumor cells escape from the cytotoxicity of radiation or chemotherapy. **(A)** Representative images of caspase-3 staining. Apoptotic cells were detected by IHC staining for caspase-3 (red) in control, irradiated, or TMZ-treated ALTS1C1 tumors. Nucleus staining with DAPI was shown in blue. **(B)** Caspase-3+ cells at tumor core and edge of each group was quantified. *n* > 33 fields per tumor of total 3 tumors in each group. **(C)** Representative figures of apoptotic cells in hypoxic or non-hypoxic region of tumors treated with irradiation or TMZ. Sections were double IHC staining for caspase-3 (red) and PIMO (green) in control, irradiated, or TMZ-treated ALTS1C1 tumors. Nucleus staining with DAPI was shown in blue. **(D)** The number of caspase-3+ cell in hypoxic or non-hypoxic region of tumors was quantified. Each group had at least three mice for data analysis. All data points were analyzed in triplicate and values displayed are means ± SDs. The significant difference of treatment groups was compared with control group and analyzed by ANOVA. ns: *P* > 0.05, *P < 0.05, **P < 0.01, ****P < 0.0001. Scale bar =50 μm.

It is well-known that hypoxic tumors are resistant to radiotherapy and chemotherapy (30–34), and we show here that brain tumors have hypoxia in tumor core as well as tumor edge. To evaluate the effects of hypoxia on treatment responses in different part of the tumor, the density of caspase-3+ cells at both regions of ALTS1C1 tumor was evaluated after radiotherapy and chemotherapy. Fourteen days after I.C injection of ALTS1C1 cells, the brain of tumor-bearing mice was treated with a dose of 15 Gy or TMZ. There was no significant difference in the density of caspase-3+ cells between the tumor edge and the tumor core after irradiation (Figures 8A,B). Similar result was also observed with the use of therapeutic drug, TMZ (Figures 8A,B). However, more caspase-3+ cells were seen in PIMO-negative, normoxic regions after radiation or TMZ treatment (core: Con vs. RT: *P* = 0.0075, Con vs. TMZ: *P* = 0.0126, edge: Con vs. RT: *P* < 0.0001, Con vs. TMZ: *P* = 0.0036, Figures 8C,D). Cells at PIMO-positive regions were barely affected by radiation or TMZ treatment. These data indicate that in addition to the hypoxia in the tumor core, the hypoxia in the tumor edge, which was not recognized before, may contribute to the resistance to cytotoxic therapy, recurrence, and invasion in high-grade glioma.

## Discussion

The brain tumor edge is frequently viewed as the tumor invasion front due to its strong invasive ability (13, 14, 17). In this study, we characterized the microenvironment at the tumor edge vs. the tumor core in a murine orthotopic astrocytoma tumor model. In agreement with our previous publication on this ALTS1C1 tumor model, the vessels in the tumor invasion front (13) or tumor edge area are denser. However, this study found no significant differences in the tumor response to radiotherapy or chemotherapy at tumor edge vs. the tumor core. This indicates that MVD is not a reliable predictor for the therapeutic resistance of tumors. A report has indicated that well-functioning tumor vessels could improve the efficacy of chemotherapy and radiotherapy by improving drug delivery and oxygenation, respectively (35). Vascular function may be more important than vessel density. This study demonstrated that the vessels at the tumor edge are heterogeneous. The transient hypoxia at the tumor edge as the result of the appearance of immature NG2-CD31+ vessels is likely one reason for the radiochemoresistance of brain tumor. Although the heterogeneity of tumor oxygenation in space and time is well-recognized, this study is the first to report two spatially and functionally distinct types of tumor hypoxia in brain tumors, namely peripheral and central hypoxia.

As cancer cells frequently proliferate faster than parenchyma, such as endothelia, tumor progression is frequently accompanied with the development of diffusion-limited hypoxia (vessel insufficiency), which leads to chronic hypoxia, or perfusion-limited hypoxia (vessel malfunction), which causes transient hypoxia. We studied the progression of hypoxia in an orthotopic brain tumor model and were surprised to find that hypoxia could be detected in the tumor as early as 3 days after tumor inoculation and throughout the period of examination (up to 21 days), despite the presence of intact, functional vessels in the brain tumors. Most of the hypoxia observed in the tumor core was caused by vascular insufficiency, but some PIMO-positive hypoxic areas began to appear at the tumor periphery in day 10 samples. Peripheral and central hypoxia could be clearly distinguished at days 14 or 21; this was further confirmed by the expression of HIF-1α and CAIX, which are several frequently-used hypoxia-associated markers that indicate different biological mechanisms of tumor hypoxia. However, we did notice that the CAIX signal was not only stronger than the other markers at the later stages (days 14 and 21), but also formed a continuous zone, unlike the discontinuous pattern of the other markers. The latter suggests either that the hypoxia at the tumor edge is transient and can be re-oxygenated, or that the tumor edge has a relatively higher metabolic rate. The appearance of PIMO-positive staining in very early small tumors indicates insufficient oxygenation at the beginning of tumor growth. Subsequently, the dominant Warburg glycolic pathway stabilizes the HIF-1α protein. At the final stage, peripheral and central regions of hypoxia can be distinguished and CAIX is overexpressed to metabolize excessive carbon dioxide.

Peripheral hypoxic tumors have been identified in glioma patients using F-fluoromisonidazole (FMISO) positron emission tomography (PET) images (36–38), but its clinical relevance has not been well-characterized. Many studies have shown that hypoxia is the main cause of tumor resistance to therapy (30–34). Here, we extend these results to peripheral hypoxia in brain tumors; our results show that tumor cells from peripheral hypoxic regions could be the main cell population that is resistant to radiation and drug therapy. These residual cells could be responsible for brain tumor recurrence and invasion after therapy (14, 39–41). It is important to note that the cells in central hypoxic regions are also resistant to therapy (41), but they will eventually be eradicated by the long-term diffusion limitation of nutrients.

This study also demonstrates that peripheral hypoxia was only observed in tumors grown in brain stroma, but not in subcutaneous or intramuscular tissues. We also found that the formation of peripheral hypoxia in intracranial tumors is independent of the tumor origin, although the degree of peripheral hypoxia varied with tumor type with the tendency of astrocytoma > glioma > melanoma. This indicates that there is a unique mechanism in cerebral tissues that causes peripheral tumor hypoxia, which may involve the interaction between glial cells, such as astrocytes and microglia and tumor cells. Although the cause of peripheral tumor hypoxia in the brain is still unclear, we noticed that the tumor border was surrounded by activated astrocytes, regardless of the type of tumor cells (Figure 6). Astrocytes are the main glial cells responsible for repairing damage in the brain, and recent reports further showed that astrocytes could be activated by ischemia, such as hypoxic brain regions (42, 43). Additionally, astrocytes can form cellular scaffolds to restore damage (44). We also found that astrocytes at the tumor border were activated at early stages (day 3; Figure 6Ba), and continued to aggregate when tumor grew up (Figures 6Bb,c). The astrocytosis patterns had good spatial correlation with peripheral hypoxia at days 14 and 21, particularly with the CAIX staining pattern. Furthermore, astrocytes overexpressed CAIX at the tumor edge, but not at the tumor core (Figure 7), indicating that there was excessive carbon dioxide in peripheral astrocytes. Astrocytic activation and metabolic alternation are usually accompanied by tumor hypoxia (42, 43, 45, 46); this theory may explain the presence of peripheral hypoxia in brain tumors. An alternative explanation is that the reactive astrocytes at the tumor edge (Figure 6Bg) introduce a compressive stress to the blood vessels (47, 48), which results in perfusion-limited hypoxia. This is supported by the observation that the vessels at PIMO-positive regions were not leaky, but were frequently obstructed (Figure 3B).

Hypoxic tumor areas are viewed as a critical region for radio- and chemoresistance (30, 31). Currently, the prevalent belief among physicians is that tumor hypoxia is generated when tumor growth outpaces vessel formation, and that hypoxia mainly occurs at the tumor core. Therefore, in clinics, higher spatial doses are used to irradiate the tumor core than the tumor edge, in order to avoid damage to normal tissue (40, 49). However, this study reports the existence of hypoxia at tumor edge, which is a distinct type of transient hypoxia in brain tumors and may escape notice during clinical examination and treatment strategy design that may result in tumor recurrence after the therapy.

## Ethics Statement

All animal experimental procedures were complied with the guideline approved by the Institutional Animal Care and Use Committee (IACUC) of National Tsing Hua University, Taiwan (IACUC: 10145).

## Author Contributions

C-ML, C-FY, and C-SC designed the study. C-ML and C-FY performed the experiments and drafted the manuscript. F-HC and J-HH designed the irradiation protocol and F-HC performed the irradiation. H-YH designed the chemotherapeutic protocol. C-FY, H-YH, J-HH, and C-SC interpreted the results. J-HH and C-SC edited and approved the manuscript.

### Conflict of Interest Statement

The authors declare that the research was conducted in the absence of any commercial or financial relationships that could be construed as a potential conflict of interest.
